# Supplementary material for: FRET biosensor uncovers cAMP nano-domains at β-adrenergic targets that dictate precise tuning of cardiac contractility
Source: Nat Commun. 2017 Apr 20;8:15031. doi: 10.1038/ncomms15031 (PMC5411486; doi:10.1038/ncomms15031)
Supplement: Supplementary Information — Supplementary Figures, Supplementary Tables, Supplementary Notes and Supplementary References [file ncomms15031-s1.pdf]

Supplementary Figures

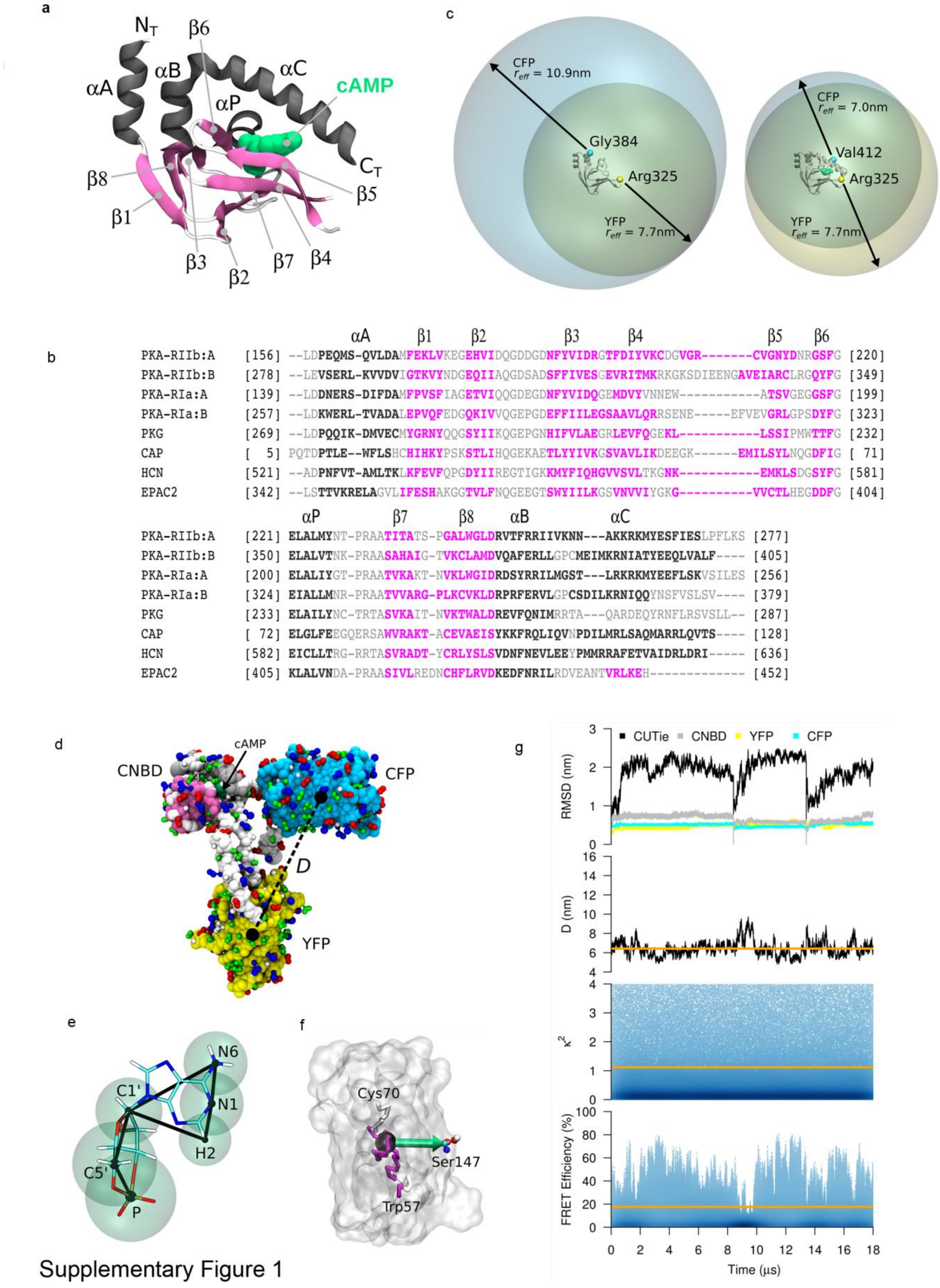

Supplementary Figure 1. Modelling and simulation of the molecular behaviour of

CUTie in the cAMP-bound state. (**a**) Cartoon diagram of the CNBD from domain B of the PKA regulatory subunit type IIβ. Structural elements are indicated by colours (grey for helix, pink for extended Beta, and white otherwise). (**b**) Sequence alignment of representative CNBDs. The alignment was generated using Clustal Omega. [1] Only CNBD domains corresponding to the following UniProt protein codes are included: PKA-RIIb domains A and B: P12369; PKA-RIa domains A and B: P00514; PKG II domain A: Q13237; CAP: P0ACJ8; HCN: O88703 and EPAC2: Q9EQZ6. Colouring of the residues corresponds to the secondary structure elements taken from the corresponding X-Ray coordinates of each protein. (**c**) Rationalization of the cAMP driven FRET sensor in terms of rough geometrical determinants. The values of r_{eff} (semitransparent spheres) indicate the maximum distance at which a fluorophore can produce FRET with a second fluorescent partner taking into account the length of the unstructured peptides connecting the CNBD with the fluorescent modules. The overlap between spheres in relation to their total volume is indicative of FRET-productive conformations. Qualitatively, the different overlapping regions between the cAMP free (left) and bound (right) conformations suggest this construction is a viable FRET sensor. It should be noted that this rough approximation does not consider important factors as excluded volume, specific interactions or relative rotations of the flourophores. [2] Panels **a** and **c** were drawn using the PDB structure 1CX4. In the cAMP free conformation (left panel), r_{eff} of CFP results from considering the 30 C-terminal amino acids (including helix C) in a completely disordered conformation, while for the r_{eff} of YFP 10 unstructured amino acids were considered to represent the linkers between the CNBD and YFP . In the cAMP bound conformation (right panel), r_{eff} of CFP is substantially shorter since only 2 C-terminal amino acids are missing in the X-ray structure the CNBD. The r_{eff} of YFP remains unchanged in relation to **a**. (**d**) Structural representation of CUTie in the cAMP-bound state at CG level. Backbone beads are coloured according to the functional domains of the sensor: CNBD (pink), YFP (yellow) and CFP (blue). Side-chain beads are shown in balls and sticks with colours of residue type: polar (green), basic (blue), acidic (red) and hydrophobic (white). Some bead radii were reduced for visualisation purposes. The inter-fluorophore distance (*D*) is indicated as well as the cAMP binding site. (**e**) Molecular representation of cAMP ligand showing its connectivity at atomistic and CG levels. Relevant atom names are indicated. (**f**) Dipole moment of the chromophore represented as a vector from the geometric center of the protein (black sphere) to the Cα atom of Ser147. The characteristic central α-helix of the GFP folding is shown as a reference. (**g**) Calculated properties along the CG simulations of CUTie in the cAMP-bound state. From top to bottom: RMSD, inter-fluorophore distance, orientational factor and FRET efficiency. Average values are reported as orange lines.

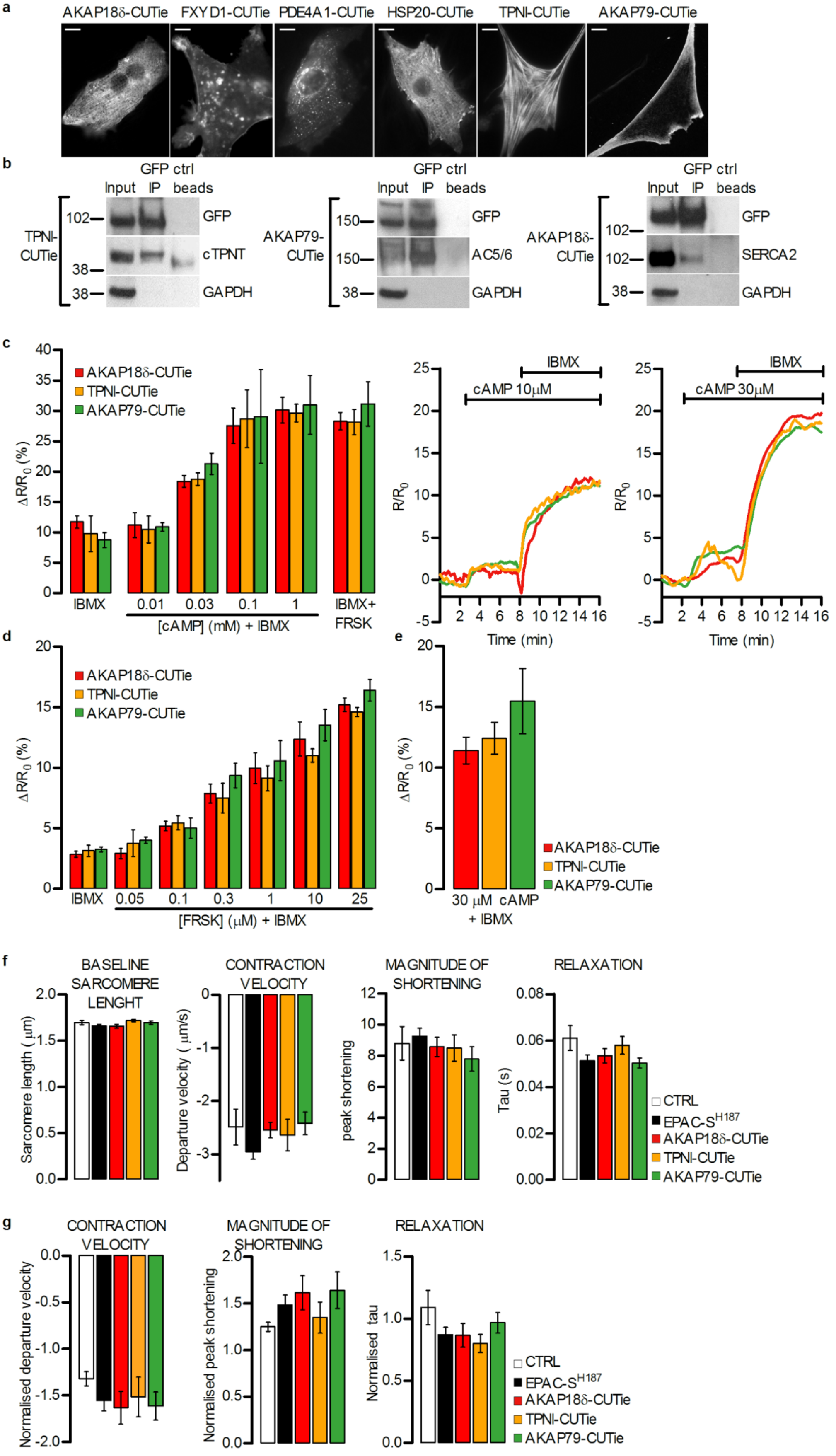

Supplementary Figure 2

Supplementary Figure 2. Localisation, calibration and effect on cardiac myocyte function of targeted CUTie chimeras. (**a**) Localisation of the targeted CUTie reporters in cultured NRVMs. Scale bar: 10 µm. (**b**) Western blotting analysis of proteins in complex with targeted CUTie. NRVMs were infected with adenovirus carrying TPNI-CUTie, AKAP18δ-CUTie or AKAP79-CUTie. Targeted CUTie chimeras and their interacting proteins were pulled down using GFP-beads. Membranes were probed with the antibodies indicated on the right of each panel. (**c**) Comparison of the FRET changes induced by the microinfusion of different [cAMP] in NRVMs, expressing targeted CUTie reporters. IBMX and FRSK+ IBMX were applied acutely in the bath. N ≥ 3. Bars indicate means ± SEM. One-way ANOVA analysis with Bonferroni post-hoc correction. Representative kinetics of FRET change on microinfusion of 10μM and 30 μM cAMP are salso shown. For representative kinetics of FRET change at saturating [cAMP] refer to **Fig 2e**. (**d**) Comparison of the FRET changes induced in ARVMs expressing targeted CUTie reporters by IBMX (100 μM) and FRSK (at the concentration indicated) + IBMX (100 μM). Bars indicate mean values ± SEM. For each concentration point N ≥ 5 from ≥ 8 biological replicates. One-way ANOVA analysis with Bonferroni post-hoc correction was applied. (**e**) Comparison of the FRET changes induced by the microinfusion of 30 μM cAMP in ARVMs expressing targeted CUTie reporters. IBMX (100 μM) was applied acutely in the bath. N = 3. Bars indicate means ± SEM. One-way ANOVA analysis with Bonferroni post-hoc correction. (**f**) Cultured ARVMs untransduced (CTRL) or transduced with adenoviral vectors carrying EPAC-S^{H187}, TPNI-CUTie, AKAP18δ-CUTie or AKAP79-CUTie were examined for sarcomere shortening. From the left: diastolic length, maximal shortening velocity (departure velocity), peak shortening (peak height as a percentage of diastolic length), and the time constant (tau) of the relaxation time. Bars indicate means ± SEM. N ≥ 10 from at least 4 biological replicates. One-way ANOVA with Bonferroni post-hoc correction. (**g**) Sarcomere shortening dynamics of cultured cells as in **d**) upon treatment with 1 nM ISO. From the left: maximal shortening velocity (departure velocity), peak shortening (peak height as a percentage of diastolic length) and the time constant (tau) of the relaxation time. To allow comparison all values are normalised to the untreated control and expressed as mean ± SEM. N ≥ 6 cells from 4 animals. One-way ANOVA with Bonferroni post-hoc correction.

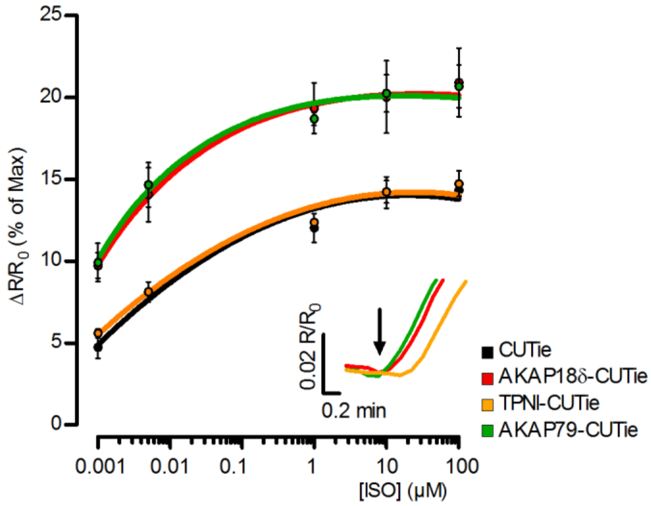

Supplementary Figure 3

Supplementary Figure 3. Comparison of amplitude and kinetics of FRET change over a range of ISO concentrations

Concentration-response curves generated by extracellular application of different ISO concentrations in cultured ARVMs expressing cytosolic CUTie, TPNI-CUTie, AKAP18δ-CUTie or AKAP79-CUTie. Data points indicate mean values ± SEM. For each concentration point N ≥ 5 from at least 4 biological replicates. For all sensors the signal is saturated at 1 μM ISO (for all sensors, $p > 0.05$ for 1 μM ISO vs 10 μM ISO and for 10 μM ISO vs 100 μM ISO). For each [ISO] the FRET response detected by AKAP18δ-CUTie or AKAP79-CUTie is significantly higher ($p < 0.05$) than the response detected by TPNI-CUTie. Inset: representative initial kinetics of FRET change on application of 10 μM ISO. Arrow indicates the time point of stimulus application.

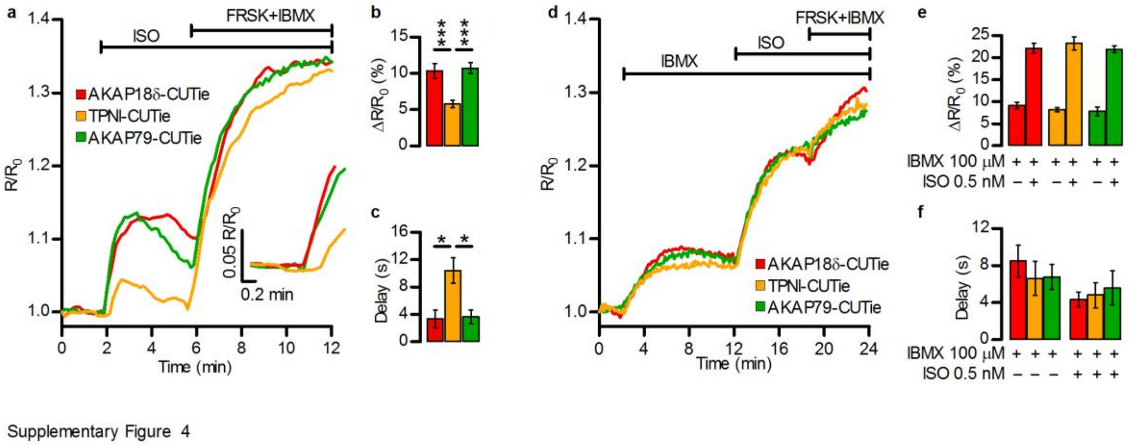

Supplementary Figure 4. cAMP signal at different β-adrenergic targets detected by targeted CUTie in NRVM. (**a**) Representative kinetics of FRET change recorded in NRVMs individually expressing AKAP18δ-CUTie, TPNI-CUTie or AKAP79-CUTie in response to bath application of 1 nM ISO and saturating stimulus. Inset: expanded time scale to illustrate the delayed response on application of ISO. (**b**) Summary of all the experiments performed as shown in **a**. N≥8. Bars indicate means ± SEM. One-way ANOVA with Bonferroni post-hoc correction. (**c**) Delay from application of 1 nM ISO to first time point at which a FRET change was detected for all experiments performed as in **a**. N ≥ 8. Bars indicate means ± SEM. (**d**) Representative kinetics of FRET change recorded in NRVM expressing AKAP18δ-CUTie, TPNI-CUTie or AKAP79-CUTie in response to bath application of 100 μM IBMX and subsequently 0.5 nM ISO. (**e**) Summary of all the experiments performed as shown in **d**. N ≥ 5. Bars indicate means ± SEM. One-way ANOVA with Bonferroni post-hoc correction. (**f**) Delay from application of 100 μM IBMX and subsequently 0.5 nM ISO to first time point at which a FRET change can be detected for all experiments performed as in **d**. For **b**, **c**, **f**, **g** N ≥ 5 from at least N = 3 biological replicates. Bars indicate means ± SEM.

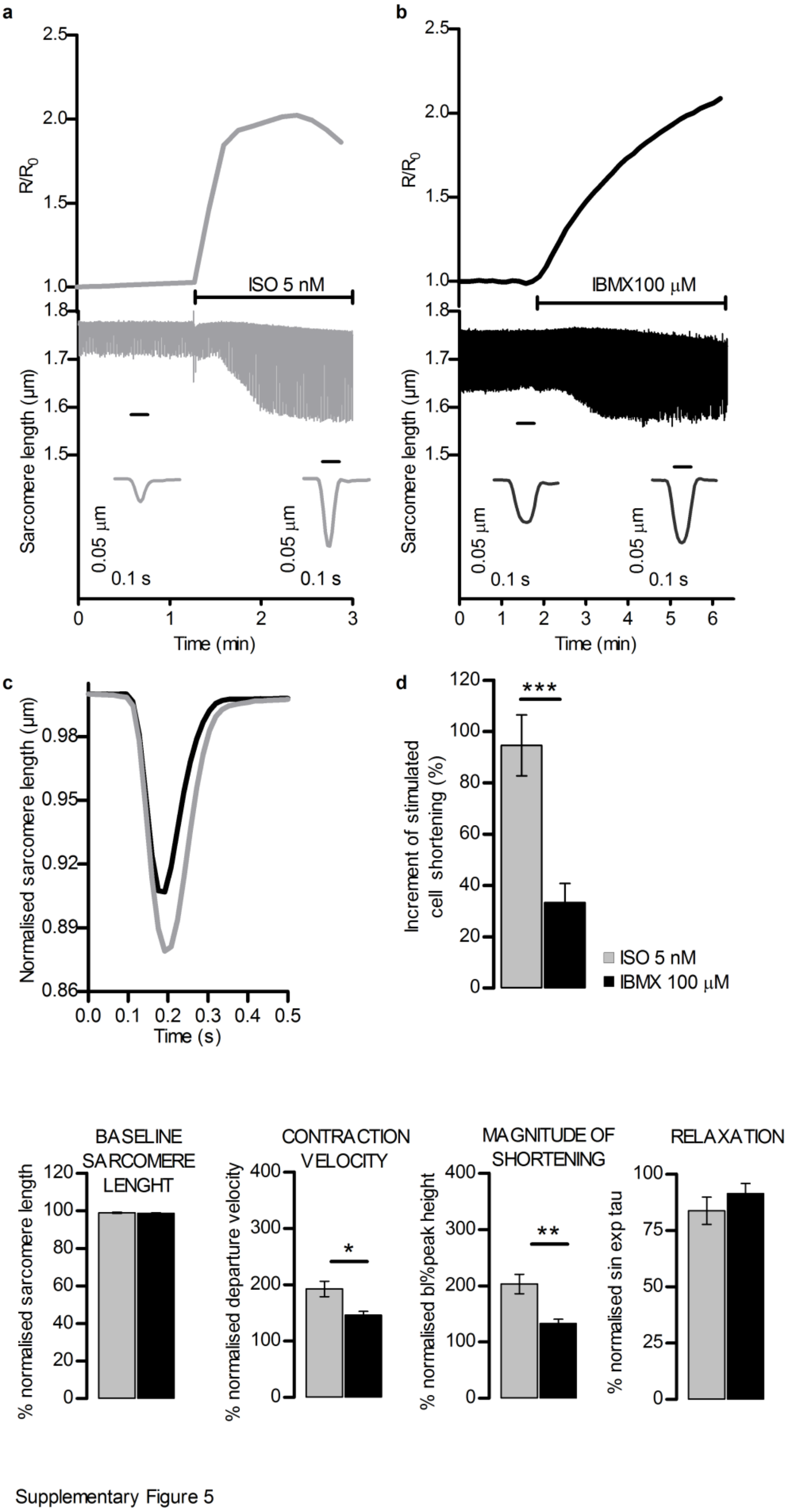

Supplementary Figure 5. Differential local regulation of cAMP signals is necessary for maximal stimulated inotropy.

(**a**) Representative time course of global cytosolic cAMP change (top) and sarcomere shortening (bottom) recorded simultaneously in the same ARVM expressing the cytosolic

FRET reporter EPAC-SH[187] on application of 5 nM ISO or (**b**) 100 μM IBMX. In **a** and **b** cells were paced at 1 Hz. Inserts at the bottom of panels **a** and **b** indicate sarcomere shortening kinetics averaged over the time interval indicated by the black bar. (**c**) Normalised mean sarcomere shortening kinetics measured at steady state after the application of ISO (5 nM) or IBMX (100 μM), as indicated. N ≥ 10. (**d**) Effect of 5 nM ISO or 100 μM IBMX on sarcomere shortening measured in all experiments as shown in **a** and **b**. Shortening is expressed as percent increment over control (before the stimulus) calculated as (Δ shortening / $shortening_{control}$) * 100, where Δ shortening = ($shortening_{stimulated}$ - $shortening_{control}$). Bars are means ± SEM, N ≥ 10. Unpaired t-test. (**e**) Summary of sarcomere shortening dynamics in cultured ARVMs on application of ISO 5 nM or IBMX 100 μM. From the left: sarcomere length, maximal shortening velocity (departure velocity), peak shortening (peak height as a percentage of diastolic length) and time constant (tau) of the relaxation time. For comparison all values were normalised to values before the treatment and expressed as percent increase. Bars indicate means ± SEM. N ≥ 10 from at least 5 biological replicates. Unpaired t-test applied.

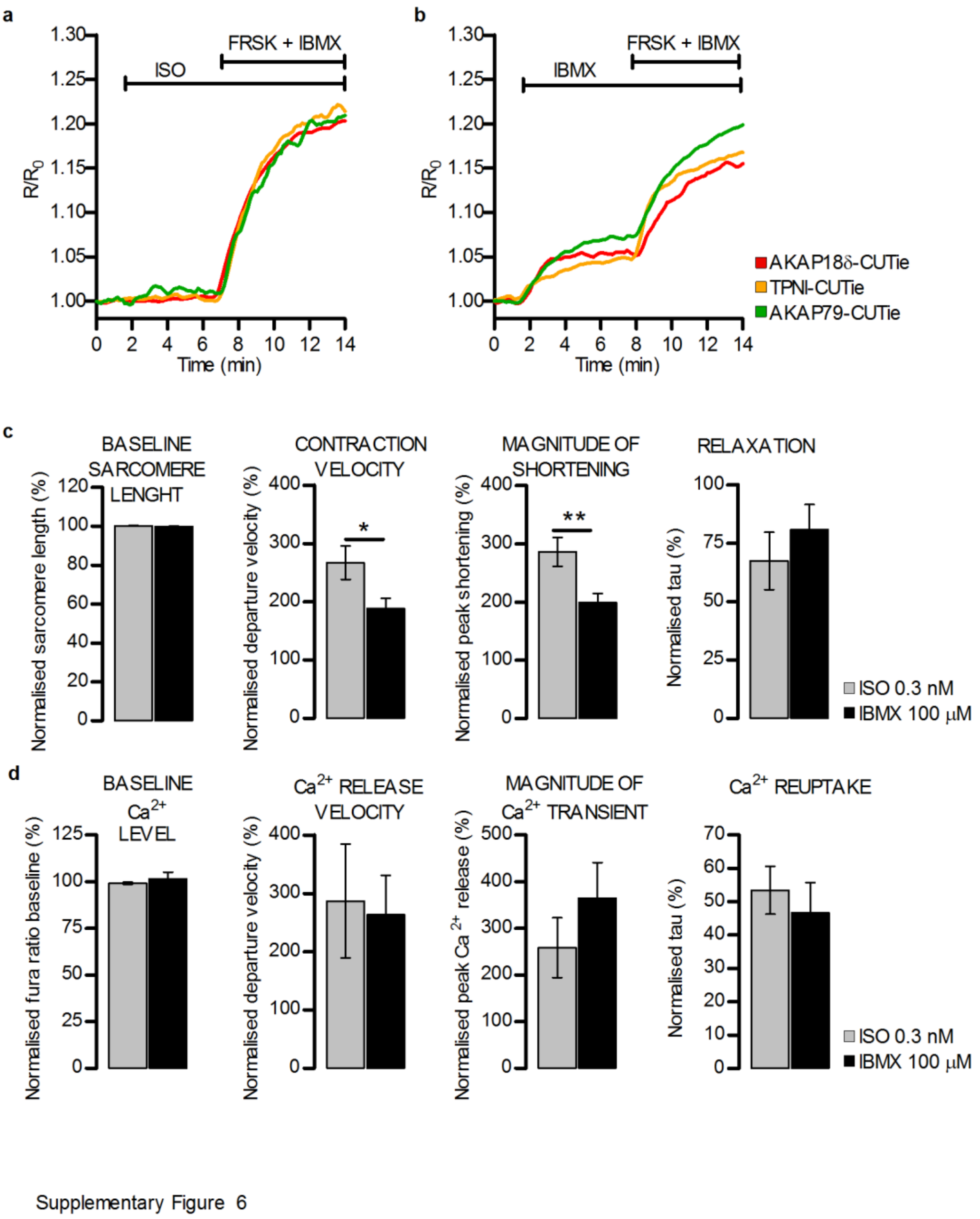

Supplementary Figure 6. Effect of compartmentalised and homogeneous cAMP rise on cardiac myocyte function. (**a**) Representative kinetics of FRET change recorded in ARLVMs expressing AKAP18δ-CUTie, TPNI-CUTie or AKAP79-CUTie in response to bath application of 0.3 nM ISO or (**b**) IBMX 100 μM and saturating stimulus. (**c**) Summary of sarcomere shortening dynamics in cultured ARVMs on application of ISO 0.3 nM or IBMX 100 μM. From the left: sarcomere length, maximal shortening velocity (departure velocity), peak shortening (peak height as a percentage of diastolic length) and time constant (tau) of the relaxation time. For comparison all values were normalised to values before the treatment and expressed as percent increase. Bars indicate means ± SEM. Unpaired t-test applied. (**d**) Summary of intracellular Ca^{2+} dynamics in cultured ARVMs on application of ISO 0.3 nM or IBMX 100 μM. From the left: resting Ca^{2+} level, maximal Ca^{2+} release velocity (departure velocity), peak Ca^{2+} release (peak height as percentage of baseline) and signal decay rate expressed as the time constant (tau) for the decay. For comparison all values were normalised to values before the treatment and expressed as percent increase. Bars indicate means ± SEM. For **c** and **d** N ≥ 6 from at least 3 biological replicates. Unpaired t-test applied.

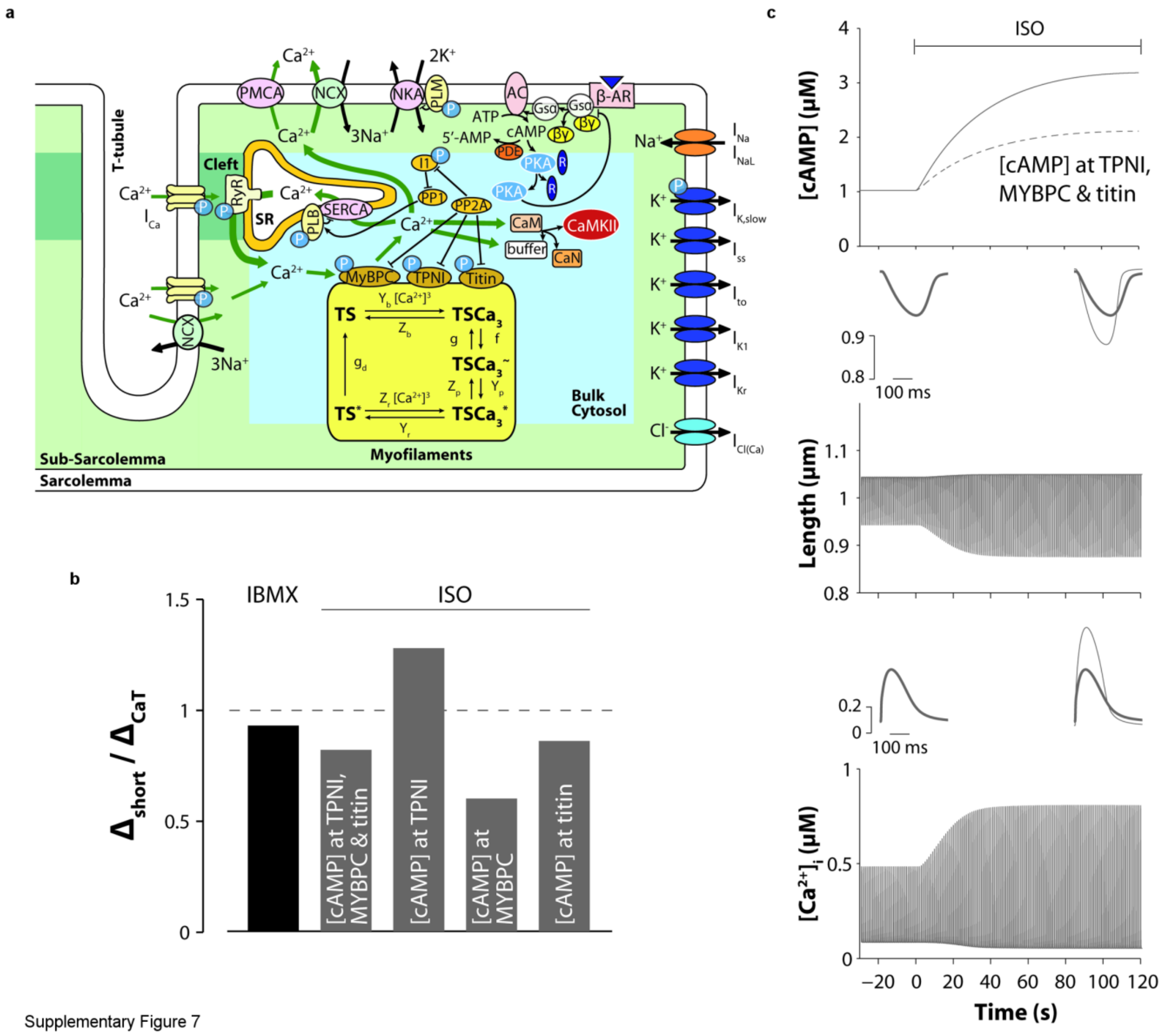

Supplementary Figure 7

Supplementary Figure 7. Model of cardiac myocyte electrophysiology, Ca^{2+}, signalling and contraction. (**a**) Schematic of our model of ionic currents, Ca^{2+} handling, cAMP-PKA-dependent signalling, and contraction in the rodent ventricular myocyte. (**b**) Bar graph showing the ratio between the increase in maximal shortening and the increase in Ca^{2+} transient amplitude induced by IBMX for IBMX and all combinations with ISO (Δ_{short} / Δ_{CaT}). (**c**) Simulated changes in myocyte shortening (middle) and Ca^{2+} transient (bottom) when cAMP rise upon ISO is reduced at all three myofilament sites (top, [cAMP] at TPNI, MyBPC & titin). Inset at the left shows baseline condition, and inset at right shows overlapping baseline condition (thick line) and steady-state response to cAMP increase. Results are presented in the main manuscript where ISO causes diminished cAMP rise at TPNI only (**Fig. 5a**, right panels).

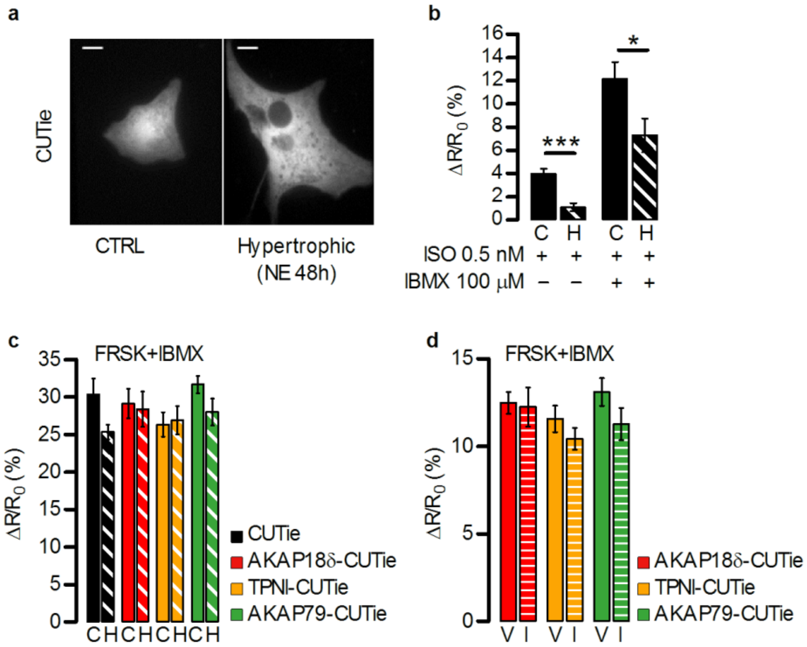

Supplementary Figure 8

Supplementary Figure 8. cAMP signalling in normal and hypertrophic myocytes. (**a**) Localisation of the cytosolic CUTie reporter in control and hypertrophic NRVMs. Scale bar: 10 µm. (**b**) Mean FRET change detected by cytosolic and CUTie reporter in control (C) and hypertrophic (H) NRVMs on application of 0.5 nM ISO alone or in the presence of 100 µM IBMX. Bars indicate means ± SEM. N ≥ 6 from at least 5 biological replicates. Unpaired t-test applied. (**c**) Mean FRET change in control and hypertrophic NRVM detected by the cytosolic and targeted CUTie reporters on application of 25 µM FRSK + 100 µM IBMX. N ≥ 6 from 5 biological replicates (**d**) Mean FRET change in ARVMs from vehicle infused (V) and ISO infused (I) rats detected by the targeted CUTie reporters on application of 25 µM FRSK + 100 µM IBMX. N ≥ 8 from at least 6 biological replicates. For **c** and **d** One-way ANOVA analysis with Bonferroni post-hoc correction shows no significant difference between all samples.

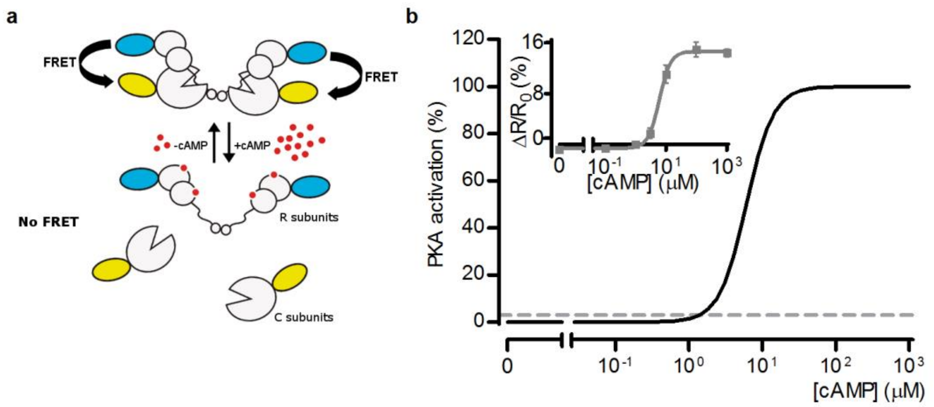

Supplementary Figure 9

Supplementary Figure 9. Determination of PKA activation level at different intracellular cAMP concentrations. (**a**) Schematic representation of the PKA-based FRET sensor[3]. In this sensor CFP is fused to the regulatory subunit (R) of PKA and YFP to the catalytic subunit (C) of PKA. At low cAMP the catalytic and regulatory subunit are associated to form the holoenzyme. In this conformation CFP and YFP are close enough for FRET to occur. When cAMP levels increase, cAMP binds to the regulatory subunits dimer and the catalytic subunits are released, abolishing FRET. Tagging of the PKA subunits with GFP has been shown not to affect the cAMP concentration-dependency activation of the enzyme. [4] Therefore this sensor, when expressed in intact cells, reliably reports the level of PKA activation in the cell. (**b**) In cell calibration curve for PKA activity generated using the PKA-based FRET sensor shown in **a** (see materials and methods for details). The FRET data used to calculate the normalised PKA activity curve are shown in the inset. For each cAMP concentration point N ≥ 5 from 3 independent experiments. Dotted line indicates basal PKA activity.

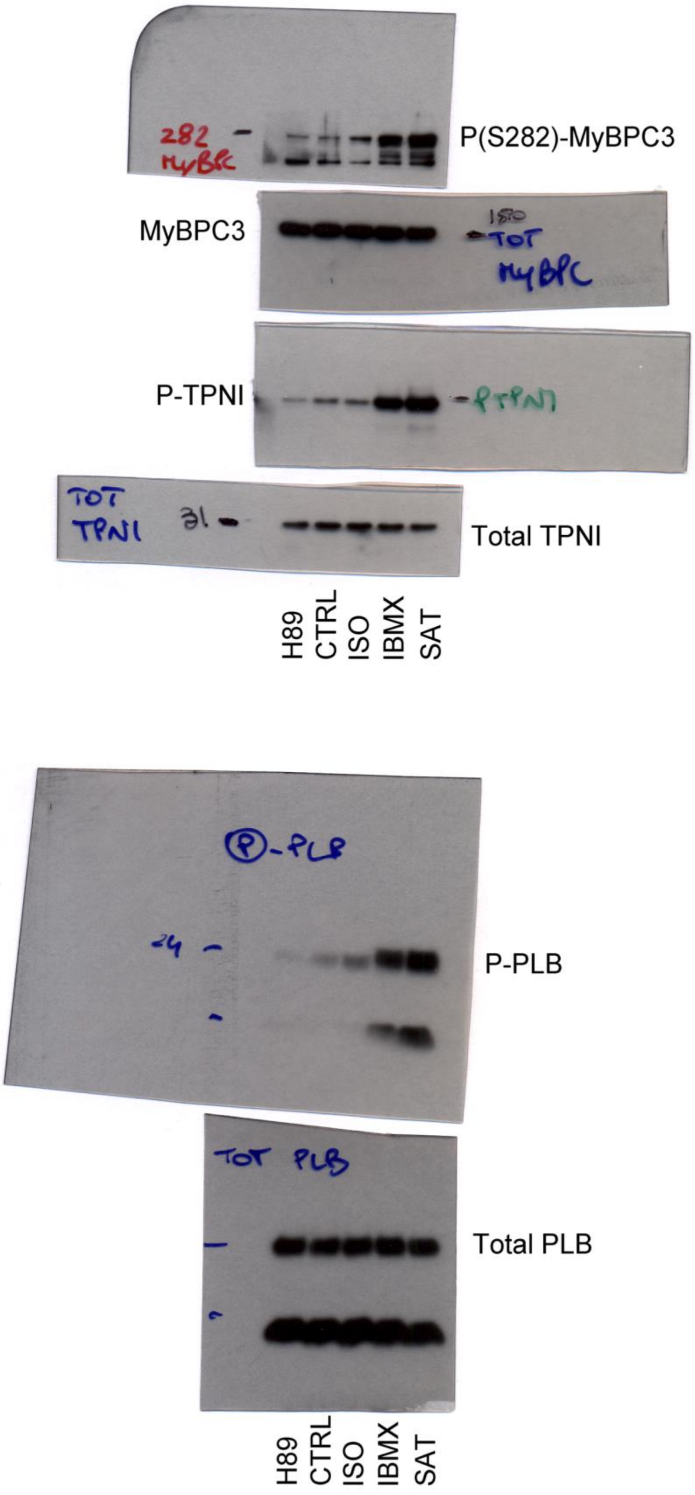

Supplementary Figure 10

Supplementary Fig 10. Uncropped scans of films showed in Figure 5. The same gel/membrane was used to probe for MyBPC and TPNI (top) and a separate gel was used to probe for PLB. Membranes were probed for phospho-protein before stripping and re-probing for the total protein.

Supplementary Tables

	Minipump infusion		Control	MI
	Vehicle	ISO		
n	5	5	6	7
IVSd	0.19 ± 0.03	0.29 ± 0.01 **		
IVSs	0.34 ± 0.04	0.45 ± 0.01 **		
LVIDd	0.60 ± 0.03	0.50 ± 0.05		
LVIDs	0.28 ± 0.02	0.13 ± 0.03 **		
LVPWd	0.23 ± 0.01	0.28 ± 0.02		
LVPWs	0.36 ± 0.01	0.48 ± 0.03 **		
EF	87.66 ± 0.98	96.53 ± 0.83 ***	84.31 ± 1.81	49.12 ± 3.60 ***
FS			54.90 ± 2.27	26.29 ± 2.27 ***
HR	355.12 ± 26.23	499.49 ± 23.31 **		
HW/BW	5.00 ± 0.40	6.25 ± 0.19 **	2.69 ± 0.03	3.10 ± 0.08 **
HW/TL	48.54 ± 4.93	60.37 ± 1.89 **	30.21 ± 0.54	37.87 ± 2.03 **

Supplementary Table 1. Characterisation of the *in vivo* models of disease.
Data are presented as means ± SEM. MI, myocardial infarction; IVSd, interventricular septum thickness – diastole (cm); IVSs, interventricular septum thickness – systole (cm); LVIDd, left ventricle internal dimension – diastole (cm); LVIDs, left ventricle internal dimension – systole (cm); LVPWd, left ventricle posterior wall thickness – diastole (cm); LVPWs, left ventricle posterior wall thickness – systole (cm); EF, ejection fraction (%); FS, fractional shortening (%); HR, heart rate (bpm); BW, body weight (g); HW, heart weight (mg); TL, tibia length (mm). Unpaired t-test was applied

**Target**	**Effect induced by PKA activation**	**Phosphatase**
LTCC	Enhanced channel opening probability and increased fraction of available channels	PP1 & PP2A
RyR	Enhanced channel opening probability	PP1 & PP2A
PLB	Enhanced Ca^{2+}-sensitivity of SERCA pump	PP1
$I_{K,slow}$	Enhanced magnitude	PP1
PLM	Enhanced Na^{+}-K^{+} ATPase activity due to increased affinity for intracellular Na^{+}	PP1
I-1	Increased activity and reduction of PP1 in the vicinity of	PP2A
TPNI	Decreased Troponin C affinity for intracellular Ca^{2+}	PP2A
MyBPC	Increased cross-bridge cycling rate	PP2A
Titin	Reduced myofilament stiffness	PP2A

Supplementary table 2. Summary of the effects of β-AR stimulation on 9 rodent-specific PKA targets and respective phosphatases

Supplementary Note 1

Design of a universal FRET-Tag for cAMP detection.

To design a novel FRET reporter for cAMP that could be used to tag proteins without affecting the efficiency of FRET we decided to use the CNBD domain B from the regulatory subunit type IIβ of PKA (PKA-RIIβ, PDB id: 1CX4) as the cAMP-sensing module. This is among the best-characterised CNBDs with X-ray structures determined in its holotetrameric (apo) and cAMP bound states.[5] This folding module (Supplementary **Fig. 1a**) shares the structural organisation of other CNBDs. [6] Its binding pocket is constituted by an 8-stranded β-barrel flanked by two helical segments commonly called helix A and B, respectively. A third helical segment, helix C, closes onto the ligand making specific contacts with it. In the absence of cAMP, helix C is often not visible from the electronic density, indicating a high mobility in its apo state.[5] This makes the C-terminus a convenient point to insert a fluorescent protein, as a significant conformational change can be expected upon cAMP binding. However, as described in the main text with the Epac1-camps sensor, inserting a FRET partner at the N-terminus may result in distortions when targeting domains are fused upstream. Hence, we sought to find a more suitable insertion point for the second FRET partner within the structure of the CNBD. Conservation analysis among CNBD sequences[7] identified loop 4-5 as the most variable region in terms of both sequence and length (Supplementary **Fig. 1b**). Indeed, this loop is not resolved in the X-ray structures of many CNBDs and previous simulations indicate this region as the most mobile within this protein module. [8, 9] Therefore, we speculated that a FRET partner could be inserted within loop 4-5 (**Fig 1c,** without perturbing the folding of neither the CNBD nor the fluorescent module. Simple geometrical considerations regarding the putative relative separation between fluorescence donor and acceptor in the cAMP bound and free conformations[2], suggest that fusion of FRET partners at the C-terminus of the CNBD and within loop 4-5 may produce a significantly higher FRET signal in the presence of cAMP (Supplementary **Fig. 1c**).

Coarse-grained simulations.

To gain more quantitative insight into the viability of the above design, 3D chimeric models were constructed on the basis of the X-ray structure of the second cAMP binding domain of PKA-RIIβ (PDB id: 1CX4) and a variant of the green fluorescent protein (PDB id: 1QYO). In all cases, the connections between protein modules involve amino acids, the coordinates of which were not resolved from the electronic density. Hence, X-ray structures of isolated protein modules were initially placed at relative distances roughly consistent with the length of unstructured amino acids in an extended conformation. The coordinates of the missing amino acids were generated using Modeller. [10] Owing to the Coarse-Grained (CG) approach used to characterise the FRET efficiency (see below), the precise determination of the unstructured regions is not highly relevant (as experiments confirmed *a posteriori*).

MD simulations suggested that the direct insertion of the fluorescent module into the 4-5 loop of the CNBD could result in structural distortions at the binding site. Hence, a spacer peptide was included to avoid structural distortions in the CNBD. A starting model of CUTie used in CG MD simulations is shown in Supplementary **Fig. 1d**.

The final sequence of the CUTie sensor, which putatively allows for the correct folding of all structural motifs, was the following:

MPFLKSLEVSERLKVVDVIGTKVYNDGEQIIAQGDSADSFFIVESGEVRITMKRKGKSPVATMVSKGEELFTGVVPILVELD
GDVNGHKFSVSGEGEGDATYGKLTLKFICTTGKLPVPWPTLVTTFGYGLQCFARYPDHMKQHDFFKSAMPEGYVQERTIFFK
DDGNYKTRAEVKFEGDTLVNRIELKGIDFKEDGNILGHKLEYNYNSHNVYIMADKQKNGIKVNFKIRHNIEDGSVQLADHYQ
QNTPIGDGPVLLPDNHYLSYQSALSKDPNEKRDHMVLLEFVTAAGITLGMDELYKSGLRSRAQASNSDIEENGAVEIARCLR
GQYFGELALVTNKPRAASAHAIGTVKCLAMDVQAFERLLGPCMEIMKRNIATYEEQLVALFGTNMDIVDVSKGEELFTGVVP
ILVELDGDVNGHRFSVSGEGEGDATYGKLTLKFICTTGKLPVPWPTLVTTLTWGVQCFSRYPDHMKQHDFFKSAMPEGYVQE
RTIFFKDDGNYKTRAEVKFEGDTLVNRIELKGIDFKEDGNILGHKLEYNYISHNVYITADKQKNGIKAHFKIRHNIEDGSVQ
LADHYQQNTPIGDGPVLLPDNHYLSTQSALSKDPNEKRDHMVLLEFVTAAGITLGMDELYK

The sequence of each functional domain is shown in a different colour: CNBD (purple), YFP (yellow) and CFP (blue). Residues that were not determined in the X-ray structures of the CNBD are shown in red and spacers in grey.

Modelling the structure, interactions and dynamics of macromolecular complexes represents an important challenge for computational biology due to the relatively large time and size scales of the processes. However, this task can be facilitated through the use of CG models, which reduce the number of degrees of freedom and allow broad exploration of complex conformational spaces[11]. Analysis of FRET variations is a particularly well-suited task for CG approaches since we are interested in the characterisation of the relative motion of fluorescent domains disregarding subtle structural details.

Here we used the SIRAH force field[12] to characterise the maximal FRET signal studying the dynamical behaviour of the CUTie sensor in a simplified fashion. The GFP variants were represented by the structure of the double mutant S65G, Y66G (PDB id: 1QYO), which retains the folding conformation in the pre-cyclization intermediate step without forming the chromophore group. Owing to the very high structural similarity between GFP variants, the same structure was used to represent YFP and CFP. This facilitated the simulation of the system at CG level without the need to parameterize the chromophore. The simplified representation of cAMP was derived from our CG model of Adenine[13] by setting P-C1'-C5' and C5'-C1'-N6 angles to 99º and 115º with a force constant of 627.6 kJ mol^{-1} rad^{-2}, while angle C5'-C1'-H2 and all dihedrals were removed (See Supplementary **Fig. 1e**).

Simulation Protocol

The simulation protocol consisted on the following steps: i) Initial all-atoms coordinates were converted to CG using SIRAH tools[14] available at http://www.sirahff.com. ii) The CG models were inserted within octahedral simulation boxes with solute-wall distance of 1.5 nm and solvated using a pre-stabilized CG water box; iii) Ionic strength was set to 150 mM of NaCl. iv) Systems underwent energy minimization; v) Equilibration MD of 5 ns were run and; vii) Production runs.

Simulations were performed using GROMACS 4.6.7 (http://www.gromacs.org) with a time step of 20 fs updating the neighbour list every 10 steps. Electrostatic interactions were calculated using Particle Mesh Ewald (PME)[15] with a direct cut off of 1.2 nm and a grid spacing of 0.2 nm. The same cut off was used for van der Waals interactions. Energy minimization was carried out by 1,000 iterations of the steepest descent algorithm. Equilibration dynamics was accomplished by 5 ns of MD with positional restraints of 1000 kJ mol^{-1} nm^{-2} applied to all the protein beads. To improve conformational sampling, three different starting conformers obtained by rotating the linker peptides were simulated in the cAMP bound conformation. Production runs for a total of 18 μs were carried out at 300 K in the NPT ensemble using the v-rescale thermostat[16] and Parrinello-Rahman barostat. [17]

Calculated Properties

Root mean square deviations (RMSD) were calculated on Cα atoms along the MD trajectories. Simulated FRET efficiency (E) was estimated from the instantaneous distances and geometrical determinants obtained from the MD simulations[18] using the relationship: $E = 1/[1 + (D/D_0)^6]$, where D is the inter-fluorophore distance and $D_0 = 9.78 \times 10^3 \times (Q_d\kappa^2\eta^{-4}J)^{1/6}$ is the Förster distance, which depends on the quantum yield (Q_d), the orientational factor (κ^2), the refractive index (η) and the overlap integral (J). In case of the YFP-CFP FRET couple in an aqueous media $Q_d = 0.42$, $\eta = 1.4$ and $J = 1.4618e^{-13}$. κ^2 was estimated as $\kappa^2 = [\sin(\theta_D)\sin(\theta_A)\cos(\phi) - 2\cos(\theta_D)\cos(\theta_A)]^2$, where angles θ_D, θ_A and ϕ describe the relative orientation between dipole moments of both fluorophores (See Figure 1 in ref[18] for more details).

Distance D was measured between geometric centre of YFP and CFP (Supplementary **Fig. 1d)**. For the calculation of κ^2, dipole-dipole angles were taken as those defined by the vectors connecting the geometric centre and the Cα atom of Ser147 in each fluorescent module (Supplementary **Fig. 1f**). This can be considered as a good approximation since it

has been shown that the dipole moment of arbitrary chromophores can be described by the line connecting the two farthest heavy atoms in the conjugated system.[19]
To estimate the maximum possible FRET signal we assumed that the cAMP free form of the sensor makes no contribution, i.e., that both fluorescent modules are not able to exchange energy because they are far apart one from each other.

The computational set up described above has already been shown to provide a very good description of cAMP binding domains and the fluorescent modules used here.[12] Hence, we will limit this section to the description of the global determinants of the FRET signal produced by CUTie in the cAMP bound state. Aimed to increase the sampling three different replicas with randomized conformation of the linkers were simulated. However, we found no significant differences in the global behaviour of the sensor, hence, the three independent trajectories are presented as a continuous one in Supplementary **Fig 1g.**
The presence of cAMP translates in a closed conformation of the binding domain with inter-fluorophore distances oscillating below 10 nm with a global average of 6.4 nm. Owing to the flexibility of the linkers used, the reciprocal orientation of both florescent modules is highly dynamic resulting in a variable FRET signal (Supplementary **Fig. 1g**). Owing to the high mobility around sigma bonds in the linkers connecting the fluorescent modules to the CNBD the time dependence of κ^2 (Supplementary **Fig. 1g**) displays a rather homogeneous distribution along all the possible values (from 0 to 4) with an average value of 1.1.
It is important to keep in mind that MD simulations can be compared to a single-molecule experiment at very high spatiotemporal resolution, while the experimental data from fluorescent microscopy measures an ensemble of conformations from a large number of molecules. Hence, in order to better compare the results from the simulation with those from cell expressing the cytosolic version of the sensor, we averaged the results obtained from the CG simulations. The FRET change on cAMP binding calculated from the simulations is 19%, in good agreement with the experimental results (**Fig. 1e**).

Supplementary Note 2

Mathematical modelling and simulation

Source codes for the entire model used to generate each figure and result in the paper is freely available online at: https://somapp.ucdmc.ucdavis.edu/Pharmacology/bers/. The present work was performed using our mouse ventricular myocyte model previously described in detail.[20] This comprehensive model includes mathematical description of the main ionic currents underlying the mouse action potential, as well as compartmentalised Ca^{2+} and Na^{+} handling, in a cellular framework laid out in the Shannon-Bers rabbit model.[21] To incorporate contractile function, we integrated our recent detailed model of myofilament contraction[22] into this mouse model, exactly as we had done for the rabbit model. No parameter or equation changes were made to the original myofilament formulation by Negroni et al.[22] Supplementary **Fig. 7a** shows the structural, ionic and myofilament framework of the model, including phosphorylation targets. When studying contractions without external load (as done in the simulations shown here), myofilament length changes depend on $[Ca^{2+}]_i$, but sarcomere length and tension also influence myofilament Ca^{2+} buffering properties. The comprehensive functionally integrated model was validated against measured changes in AP, $[Ca^{2+}]$, and $[Na^{+}]$ in response to ISO. Notably, coupling the electrophysiological and contractile systems did not affect overall electrophysiological or Ca^{2+} handling behaviour of the model, which recapitulates previous results validated against a broad range of experimental data.[20]

The cellular model also includes biochemically-detailed description of PKA and Ca^{2+}/calmodulin-dependent protein kinase II (CaMKII) signalling pathways, adapted from the original formulation developed by Soltis and Saucerman[23] and our further modifications in our core mouse model.[20] In our prior work we showed that CaMKII target regulation had little impact on PKA-dependent changes in electrophysiological, Ca^{2+} handling and contraction. As shown in Supplementary **Fig. 7a**, our formulation describes agonist stimulation of β1-adrenergic receptor (β-AR), activation of the stimulatory G protein α-subunit ($G_s\alpha$), cAMP synthesis by AC and degradation by PDE, PKA subunit activation and dissociation, PKA-mediated β-AR desensitisation and phosphorylation of different target proteins (including three myofilament proteins, see below), and target dephosphorylation by protein phosphatase-1 (PP1) and/or protein phosphatase-2A (PP2A). In the baseline formulation, AC and PDE are uniformly distributed within the cell, and thus cAMP and PKA rise uniformly upon β-adrenergic stimulation (i.e., cAMP signal is the same at each target). We revised the model to simulate diminished cAMP rise at certain contractile targets upon ISO administration. Namely, we simulated reduced [cAMP] at TPNI, MyBPC & titin (Supplementary **Fig. 7c**), at TPNI only (**Fig. 5a**, right panels), at MYBPC only, and at titin only (Supplementary **Fig. 7b**).

Supplementary table 2 summarizes the effects of β-AR stimulation on 9 rodent-specific targets, which include: LTCC, ryanodine receptors (RyR), PLB, slowly inactivating K^{+} current ($I_{K,slow}$), phospholemman (PLM), Inhibitor-1 (I-1), TPNI, MyBPC, and titin. Respective phosphatases are represented as well. Quantitative PKA-dependent changes in these properties are the same as explicitly described in Morotti et al.[20] (ionic and Ca^{2+} handling), with no parameter or equation changes compared to the original formulation. In the parent models,[24 25, 26 23 20] both biochemical data and functional readouts from the literature (generally measured in vitro with purified components or measured in ventricular myocytes) were used for parameterization of the phosphorylation and dephosphorylation rates and phosphorylation effects at any given target. When kinetic data were not available,

parameters were estimated based on data from similar proteins (e.g., with same localisation within the cell), as previously described.[23-26]

Here, dynamic PKA-dependent phosphorylation of myofilament proteins (TPNI, MyBPC, and titin) was implemented as previously described,[22] with phosphorylation extent and kinetics used for TPNI in the Morotti et al. model.[20] We assumed the same total concentration as well (70 µmol/L cytosol). ISO administration reduces myofilament stiffness and Ca^{2+}sensitivity (XBCa), and increases crossbridge cycling rate (XBCy). The following terms refer to the fractional level of PKA-dependent phosphorylation of myofilament targets

$$fracPKA\_Myo0 \ = \ 0.062698\,; \ \ [derived\ quantity]$$
$$fracPKA\_Myoiso \ = \ 0.868762\,; \ \ [derived\ quantity]$$

Where $fracPKA\_Myo0$ is the baseline level of fractional phosphorylation at 1 Hz stimulation without ISO, and $fracPKA\_Myoiso$ is the maximal level achieved with 100 nM ISO. The following equation uses the current value of fractional phosphorylation ($fracPKA\_Myo$) to scale the ISO effect linearly on myofilament parameters.

$$kPKA\_Myo \ = \ \frac{fracPKA\_Myo \ - \ fracPKA\_Myo0}{fracPKA\_Myoiso \ - \ fracPKA\_Myo0}$$

The myofilament parameter equations below (as in Negroni et al.) are mostly reflected in the diagram in Supplementary **Fig. 7a**. These are clustered for effects on stiffness Myo (attributed to titin, $\boldsymbol{K_e}$), myofilament Ca^{2+} sensitivity $XBCa$ (attributed to TPNI, $\boldsymbol{Z_b}$-$\boldsymbol{Y_r}$) and crossbridge cycling $XBCy$ (attributed to MyBPC, $\boldsymbol{Z_a}$-$\boldsymbol{Y_v}$). This allows separate control of effects at titin, TPNI and MyBPC.

$$K_e = (1 + uMyo\,(0.5 - 1)\ kPKA\_Myo)\ 105000\,; [mN\ mm^{-2}\ \mu m^{-5}]$$
$$Z_b = (1 + uXBCa\,(4.2 - 1)\ kPKA\_Myo)\ 0.1397\,; [ms^{-1}]$$
$$Z_r = (1 + uXBCa\,(1.8 - 1)\ kPKA\_Myo)\ 7262.6e6\,; [mM^{-3}\ ms^{-1}]$$
$$Y_r = (1 + uXBCa\,(2.2 - 1)\ kPKA\_Myo)\ 0.1397\,; [ms^{-1}]$$
$$Z_a = (1 + uXBCy\,(1.24 - 1)\ kPKA\_Myo)\ 0.0023\,; [ms^{-1}]$$
$$f = (1 + uXBCy\,(1.24 - 1)\ kPKA\_Myo)\ 0.0023\,; [ms^{-1}]$$
$$RLa \ = (1 + uXBCy\,(0.4 - 1)\ kPKA\_Myo)\ 20\,; [\mu m^{-2}]$$
$$Z_p \ = (1 + uXBCy\,(2.2 - 1)\ kPKA\_Myo)\ 0.2095\,; [ms^{-1}]$$
$$Y_p \ = (1 + uXBCy\,(2.2 - 1)\ kPKA\_Myo)\ 0.1397\,; [ms^{-1}]$$
$$B_p \ = (1 + uXBCy\,(3.4 - 1)\ kPKA\_Myo)\ 0.5\,; [ms^{-1}]$$
$$B_w = (1 + uXBCy\,(3.4 - 1)\ kPKA\_Myo)\ 0.35\,; [ms^{-1}]$$
$$Y_c \ = (1 + uXBCy\,(0.4 - 1)\ kPKA\_Myo)\ 4\,; [\mu m^{-1}]$$
$$Y_d \ = (1 + uXBCy\,(2.2 - 1)\ kPKA\_Myo)\ 0.028\,; [ms^{-1}]$$
$$Y_v \ = (1 + uXBCy\,(1.6 - 1)\ kPKA\_Myo)\ 0.9\,. [ms^{-1}]$$

Where *uMyo*, *uXBCa*, and *uXBCy* are 1 in IBMX simulations; with ISO, *uMyo*, *uXBCa*, and *uXBCy* are 0.5 when cAMP rise is limited at all three myofilament sites ([cAMP] at TPNI, MyBPC & titin), *uMyo*, *uXBCa* are 1 and *uXBCy* is 0.5 when cAMP rise is reduced at MyBPC only, *uMyo*, *uXBCy* are 1 and *uXBCa* is 0.5 when cAMP rise is reduced at TPNI only, and *uMyo* is 0.5 and *uXBCa* and *uXBCy* are 1 when cAMP rise is reduced at titin only. Thus, to simulate diminished [cAMP] at a given target, we scaled the functional target effect of PKA

by 50% (described in the table) by reducing the change induced in the affected model parameters. In the case of titin, for example, the parameter K_e describing myofilament stiffness was reduced from 105,000 to 52,500 when simulating uniform cAMP rise within the cell, and to 78,750 (in mN mm^{-2} μm^{-5}) when simulating diminished cAMP signal.

Supplementary References

1. Sievers, F. *et al.* Fast, scalable generation of high-quality protein multiple sequence alignments using Clustal Omega. *Molecular systems biology* **7**, 539 (2011).
2. Machado, M. & Pantano, S. Structure-based, in silico approaches for the development of novel cAMP FRET reporters. *Methods Mol Biol* **1294**, 41-58 (2015).
3. Zaccolo, M. *et al.* A genetically encoded, fluorescent indicator for cyclic AMP in living cells. *Nat Cell Biol* **2**, 25-29 (2000).
4. Mongillo, M. *et al.* Fluorescence resonance energy transfer-based analysis of cAMP dynamics in live neonatal rat cardiac myocytes reveals distinct functions of compartmentalized phosphodiesterases. *Circ Res* **95**, 67-75 (2004).
5. Zhang, P. *et al.* Structure and allostery of the PKA RIIbeta tetrameric holoenzyme. *Science* **335**, 712-716 (2012).
6. Berman, H.M. *et al.* The cAMP binding domain: an ancient signaling module. *Proc Natl Acad Sci U S A* **102**, 45-50 (2005).
7. Canaves, J.M. & Taylor, S.S. Classification and phylogenetic analysis of the cAMP-dependent protein kinase regulatory subunit family. *Journal of molecular evolution* **54**, 17-29 (2002).
8. Berrera, M., Pantano, S. & Carloni, P. cAMP Modulation of the cytoplasmic domain in the HCN2 channel investigated by molecular simulations. *Biophysical journal* **90**, 3428-3433 (2006).
9. Berrera, M., Pantano, S. & Carloni, P. Catabolite activator protein in aqueous solution: a molecular simulation study. *The journal of physical chemistry. B* **111**, 1496-1501 (2007).
10. Eswar, N. *et al.* Comparative protein structure modeling using MODELLER. *Current protocols in protein science / editorial board, John E. Coligan ... [et al.]* **Chapter 2**, Unit 2 9 (2007).
11. Ingolfsson, H.I. *et al.* The power of coarse graining in biomolecular simulations. *Wiley interdisciplinary reviews. Computational molecular science* **4**, 225-248 (2014).
12. Darre, L. *et al.* SIRAH: A Structurally Unbiased Coarse-Grained Force Field for Proteins with Aqueous Solvation and Long-Range Electrostatics. *Journal of chemical theory and computation* **11**, 723-739 (2015).
13. Dans, P.D., Zeida, A., Machado, M.R. & Pantano, S. A Coarse Grained Model for Atomic-Detailed DNA Simulations with Explicit Electrostatics. *Journal of chemical theory and computation* **6**, 1711-1725 (2010).
14. Machado, M. & Pantano, S. SIRAH Tools: mapping, backmapping and visualization of coarse-grained models. *Bioinformatics* (2016).
15. Darden, T., York, D. & Pedersen, L. Particle Mesh Ewald - an N.Log(N) Method for Ewald Sums in Large Systems. *Journal of Chemical Physics* **98**, 10089-10092 (1993).
16. Bussi, G., Donadio, D. & Parrinello, M. Canonical sampling through velocity rescaling. *The Journal of chemical physics* **126**, 014101 (2007).
17. Parrinello, M. & Rahman, A. Polymorphic Transitions in Single-Crystals - a New Molecular-Dynamics Method. *J Appl Phys* **52**, 7182-7190 (1981).
18. Pantano, S. In silico description of fluorescent probes in vivo. *J Mol Graph Model* **27**, 563-567 (2008).
19. Ansbacher, T. *et al.* Calculation of transition dipole moment in fluorescent proteins--towards efficient energy transfer. *Physical chemistry chemical physics : PCCP* **14**, 4109-4117 (2012).
20. Morotti, S., Edwards, A.G., McCulloch, A.D., Bers, D.M. & Grandi, E. A novel computational model of mouse myocyte electrophysiology to assess the synergy between Na+ loading and CaMKII. *The Journal of physiology* **592**, 1181-1197 (2014).
21. Shannon, T.R., Wang, F., Puglisi, J., Weber, C. & Bers, D.M. A mathematical treatment of integrated Ca dynamics within the ventricular myocyte. *Biophysical journal* **87**, 3351-3371 (2004).

22. Negroni, J.A. *et al.* beta-adrenergic effects on cardiac myofilaments and contraction in an integrated rabbit ventricular myocyte model. *Journal of molecular and cellular cardiology* **81**, 162-175 (2015).
23. Soltis, A.R. & Saucerman, J.J. Synergy between CaMKII substrates and beta-adrenergic signaling in regulation of cardiac myocyte Ca(2+) handling. *Biophysical journal* **99**, 2038-2047 (2010).
24. Saucerman, J.J., Brunton, L.L., Michailova, A.P. & McCulloch, A.D. Modeling beta-adrenergic control of cardiac myocyte contractility in silico. *The Journal of biological chemistry* **278**, 47997-48003 (2003).
25. Saucerman, J.J. & McCulloch, A.D. Mechanistic systems models of cell signaling networks: a case study of myocyte adrenergic regulation. *Progress in biophysics and molecular biology* **85**, 261-278 (2004).
26. Saucerman, J.J., Healy, S.N., Belik, M.E., Puglisi, J.L. & McCulloch, A.D. Proarrhythmic consequences of a KCNQ1 AKAP-binding domain mutation: computational models of whole cells and heterogeneous tissue. *Circ Res* **95**, 1216-1224 (2004).
